# Sleep Aid Effect and Mechanism of Semen Zizyphi Spinosae Extract Enriched With Jujuboside and Jujubogenin in Sleep‐Deprived Zebrafish

**DOI:** 10.1002/fsn3.70413

**Published:** 2025-06-11

**Authors:** Wanxia Wang, Can Li, Lu Shen, Yifan Liu, Jie Zhou, Chengyun Zheng, Dongqi Tang, Fang Xiao, Tao Xia

**Affiliations:** ^1^ School of Bioengineering Qilu University of Technology (Shandong Academy of Science) Jinan Shandong P. R. China; ^2^ State Key Laboratory of Biobased Material and Green Papermaking Qilu University of Technology (Shandong Academy of Science) Jinan Shandong P. R. China; ^3^ Department of Hematology, the Second Hospital, Cheeloo College of Medicine Shandong University Jinan Shandong P. R. China; ^4^ Center for Gene and Immunotherapy, Multidisciplinary Innovation Center for Nephrology, the Second Hospital, Cheeloo College of Medicine Shandong University Jinan Shandong P. R. China; ^5^ Department of Cadres Medical Care and Gerontology, the Second Hospital, Cheeloo College of Medicine Shandong University Jinan Shandong P. R. China

**Keywords:** β‐Glucosidase, GABAergic, HCRTergic, Jujubogenin, Jujuboside, NMU, semen ziziphi spinosae, sleeping aid effect

## Abstract

The jujuboside in semen zizyphi spinosae has various biological activities beneficial to human health, but its natural content is relatively low, which limits its application in biomedicine and health care. This study employed ultrasonic‐assisted alcohol extraction to isolate jujuboside from semen zizyphi spinosae, and the content significantly increased by 482.14%. By treating the extract with β‐glucosidase, the content of jujubogenin increased by 6.34%. The extract treated with β‐glucosidase had a better sleep improvement effect (86.54%) on sleep‐deprived zebrafish than the untreated extract (44.64%). Its sleep improvement effect was 32.24% and 34.99% of γ‐aminobutyric acid (GABA) and estazolam, respectively, and did not show any drowsiness similar to the positive controls. The extracts effectively restored the daytime awakening activity of sleep‐deprived zebrafish and notably increased levels of GABA, hypocretin/orexin (HCRT), and neuromedin U (NMU). The sleep‐regulatory mechanisms of jujubogenin and jujuboside in the extracts of semen zizyphi spinosae may be associated with the expression of GABA receptor subtypes and related genes in GABAergic, HCRTergic, and NMU systems. The research findings indicated that enrichment of jujuboside in semen zizyphi spinosae and utilization of β‐glucosidase to further enhance the content of jujubogenin are of great significance for the treatment of insomnia and promoting rehabilitation.

## Graphical abstract

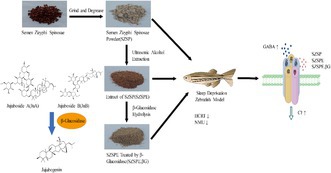

**Abbreviations**
JuA: Jujuboside A
JuB: Jujuboside B
SZSP: Semen Zizyphi Spinosae Powder
SZSPE: Extract of SZSP
SZSPE.βG: SZSPE Treated by β‐ Glucosidase

## 1 Introduction

Insomnia is a complex physico‐psychological condition that results from various factors, including environmental changes and physical or mental illnesses (Liu et al. 2019). It is a sleep disorder and one of the most common clinical complaints, characterized by persistent difficulties in falling asleep, maintaining sleep, or achieving nonrestorative sleep, ultimately leading to inadequate sleep satisfaction and accompanied by significant daytime symptoms such as fatigue, decreased concentration, impaired cognitive functioning, irritability, anxiety, and depressed mood (Riemann et al. 2022). Insomnia can disrupt the functioning of the body's autonomic nervous system, giving rise to negative emotions such as tension, anxiety, depression, compulsion, and even cognitive decline, memory impairment, and severe mental disorders like hallucinations and delusions. Persistent insomnia is very likely to lead to the elevation of blood glucose, blood lipids, and blood pressure, which triggers cardiovascular and cerebrovascular complications (Ford and Kamerow 1989; Peng 2014). Around 30% of people worldwide have one or more insomnia symptoms (Roth 2007), and a survey by the China Sleep Research Society in 2021 showed that the prevalence of insomnia among adults in China is as high as 50.41%.

Traditional approaches for managing insomnia encompass cognitive behavioral therapy (CBT) and pharmaceutical interventions. Although the American College of Physicians has identified CBT as the first‐line treatment for insomnia (Koffel et al. 2018), it has drawbacks such as high cost, long consultation time, lack of trained providers, and inability to achieve immediate results, making it difficult to implement in clinical practice (Aoki et al. 2022). Hypnotic drugs are considered necessary in many cases and continue to be widely used (Madari et al. 2021). Barbiturates are the earliest used sedative‐hypnotic drugs but have been largely phased out in clinical practice due to their safety issues (Du et al. 2022). Currently, benzodiazepines (BZ) and non‐benzodiazepine (NBZ) are widely used in clinical practice. After their binding with the receptors of GABA or GABA subtypes, the chloride channel undergoes a conformational change, leading to hyperpolarization and subsequent inhibition of the central nervous system to exert a sedative‐hypnotic effect (Kelly et al. 2002; Xu et al. 2014). Long‐term use of other categories of drugs, such as melatonin, sedative antidepressants, and orexin receptor antagonists, can also lead to many side effects, including cognitive impairment, tolerance, rebound insomnia after discontinuation, abuse, dependence liability, and even respiratory depression (Atkin et al. 2018; De Crescenzo et al. 2022; Osamu et al. 1993).

Numerous studies have demonstrated that natural drugs and extracts offer distinct benefits in improving both the quantity and quality of sleep. Various chemical components contained in plants, such as saponins, terpenoids, flavonoids, alkaloids, steroids, endocannabinoids, cannabinoid cinnamates, nitrites, valerylates, and cyclic enol ethers, have exhibited the potential to improve sleep by modulating the GABAergic system, 5‐HTergic system, melatonin neurotransmitters, and histamine neurotransmitters. Moreover, these natural compounds are cost‐effective, efficient, and associated with fewer side effects, making them a promising alternative therapy (Akram et al. 2019; Bruni et al. 2021).

Semen zizyphi spinosae is the dried mature seeds of *Ziziphus jujuba Mill. var. spinosa (Bunge) Hu ex H. F. Chou*, belonging to the Rhamnaceae family, which is widely distributed in Asia, Europe, and Australia (He et al. 2020). It is mainly planted in inland regions of northern China and is commonly used in the treatment of symptoms such as restlessness and insomnia, palpitations and excessive dreaming, excessive sweating and thirst (Gao et al. 2013; Hua et al. 2022). It has a unique effect in treating insomnia and is the preferred single herbal medicine in traditional Chinese medicine for this purpose (Zheng et al. 2006), which is also one of the earliest Chinese medicines to be categorized as homology of medicine and food (medicinal food) (Y.‐L. Chen et al. 2015). The active ingredients in semen zizyphi spinosae include jujuboside, flavonoids, alkaloids, fatty acids, etc. Among them, sour jujube kernel saponins are the main substances that exert sedative‐hypnotic effects (Du et al. 2019), with slow onset and long duration of action and no obvious addiction or tolerance (Geng and Li 2016). When taken by the human body, it ingests glycoside compounds in the form of prodrugs, which are difficult to absorb in the intestine and have low bioavailability (Zuo et al. 2002).

Saponins are a class of glycosides whose glycosides are spirostanes or triterpenoids, which are named for their ability to form stable soap‐like foam in aqueous solution and have a wide range of biological activities such as sedative, antipyretic, anti‐inflammatory, antimicrobial, antiviral, cardiovascular system protection, etc. (Liu et al. 2017). They are widely present in terrestrial higher plants, and a few marine organisms also contain saponin compounds (Moses et al. 2014; Serventi et al. 2013). Notably, saponins are prominent active constituents in various commonly used Chinese herbal medicines, including semen zizyphi spinosa, ginseng, ganoderma lucidum, and licorice (Jiang et al. 2023). The natural saponins present in plants are converted by enzymes, microorganisms, and gut microbiota to form secondary glycosides or aglycones. These derivatives typically involve the removal of sugar groups, resulting in reduced polarity and increased liposolubility. After ingestion by the human body, these modified saponins can be rapidly absorbed through the small intestine, enter the bloodstream, and efficiently attain the necessary blood concentration to manifest their pharmacological effects (Silberberg et al. 2006). Converting glycosides into more active aglycones through biotransformation can improve their bioavailability and is crucial for their pharmacological effect.

Enzymatic conversion reaction has become a promising method for producing aglycones, primarily due to its mild reaction conditions, high specificity, non‐destructive effect on the core structure of saponins, and fewer by‐products. Among the various enzymatic reactions, β‐glucosidase has emerged as one of the most extensively studied enzymes for saponin transformation (Otieno et al. 2006). This enzyme catalyzes the hydrolysis of glycoside compounds, leading to the formation of functional aglycones and exhibiting heightened biological activities. For instance, the utilization of β‐glucosidase can facilitate the hydrolysis of steviol glycosides, resulting in the production of more bioactive hydrolysis products, such as steviol glycosides, steviol alcohols, isosteviol alcohols, and steviol monoglycosides (Chen and Li 2009). Additionally, studies have demonstrated that β‐glucosidase can convert ginsenoside Rg3 to the rare saponin Rh2 by cleaving the glycosidic bond at the terminal C‐3 position (Pei et al. 2015). Despite these advancements, the application of β‐glucosidase in extracting and converting jujuboside from semen zizyphi spinosa remains unexplored, with no existing reports detailing its study and practical implementation.

In this study, β‐glucosidase was applied to the extraction process of jujuboside from semen zizyphi spinosa in order to obtain jujubogenin with higher biological activity. Through the behavioral drug screening platform of zebrafish, we carried out the verification of sleep‐aiding function and investigated the biochemical and molecular mechanisms of its sleep‐improving effect, so as to provide new ideas and methods for the efficient transformation of jujuboside as well as the prevention and treatment of sleep disorders.

## 2 Materials and Methods

### 2.1 Preparation of Semen Zizyphi Spinosae Powder (SZSP), Extract of SZSP (SZSPE), SZSPE Treated by β‐Glucosidase (SZSPE.βG)

The material in this study was semen zizyphi spinosa which was purchased from Xingtai City, Hebei Province, China.

Preparation of Semen Zizyphi Spinosae Powder (SZSP): Semen zizyphi spinosa is dried, and an appropriate amount is taken into a pulverizer to pulverize and passed through a 40‐mesh sieve.

Preparation of Extract of SZSP (SZSPE): An appropriate amount of sieved SZSP is taken, and put in a Soxhlet extractor, and degreased it by refluxing with 6 times the amount of petroleum ether at 60°C–90°C for two times, each time for 2 h, until the reflux liquid became colorless. The petroleum ether extract was discarded and placed it on a drying oven at 60°C for drying. After drying, a ball mill was used to crush it into powder (Sun et al. 2019). An appropriate amount of defatted SZSP was mixed with 70% ethanol at a material–liquid ratio of 1:30 and extracted by ultrasonic waves for 30 min under the condition of 40°C and ultrasonic power of 180 W. It was centrifuged at 4°C and 5000 r/min for 15 min, and then the supernatant was taken at a constant temperature of 50°C–60°C for rotary evaporation; the concentrated solution was dried into powder in a blast drying oven at 50°C.

Preparation of samples of SZSPE Treated by β‐glucosidase (SZSPE.βG): 100 mg of β‐glucosidase (300 U/g; Solarbio Technology Co., LTD, Beijing, China) was precisely weighed, dissolved in citric acid–sodium citrate buffer at pH 5.0, and fixed to 300 mL. 5 g of SZSPE was weighed and dissolved in 50 mL of β‐glucosidase buffer, shook well at 50°C and 200 rpm for 12 h, and then dried.

The total jujuboside content of each sample was determined by the vanillin glacial acetic acid colorimetric method (Zhao and Shu 2013).

### 2.2 Liquid Mass Target Quantification of Jujuboside A (JuA) and Jujuboside B (JuB) in SZSP, SZSPE, and SZSPE.βG

A 0.10 g of sample was accurately weighed, 1.0 mL of methanol was added, shook well, and ultrasonically extracted at room temperature for 30 min, centrifuged at 3500 r/min, and the supernatant was absorbed. 1.0 mL of methanol was added to the filter residue, and the extraction was repeated two times, and then the three extracts were combined. After filtering with a 0.22 μm filter membrane, it is stored in the dark at 4°C, and diluted 10 times with a 50% methanol aqueous solution before analysis.

The standard (5 mg) is dissolved in a 1 mL volumetric flask, and a 5 mg/mL standard storage solution is prepared. The standard solution is diluted step by step to obtain a series of calibration solutions.

An Agilent 1260–6470 liquid chromatography–triple quadrupole mass spectrometry system and Agilent C18 chromatographic column (2.1 × 50 mm, 1.8 μm) were used for analysis (Agilent Technology Co. Ltd., Beijing, China). Injection volume was 5 μL. The mobile phase A was a 0.1% formic acid aqueous solution and B was acetonitrile. Elution gradient: 0–1 min 10% B, 1–5 min 10%–90% B, 5.1–8 min 10% B, flow rate of 0.4 mL/min. Mass spectrometry analysis was performed in multiple reaction monitoring (MRM) mode using an Agilent 6470 triple quadrupole mass spectrometer equipped with an ESI ion source. Ion source parameters: ESI (−), electric spray voltage 4000 V, ion source temperature 350°C, atomization pressure 30 psig, dry gas flow rate 10 L/min.

### 2.3 Preparation of Zebrafish Sleep Deprivation Model and Grouping of Each Treatment

Zebrafish were raised in the Zebrafish Laboratory of Suzhou Murui Biotech Co. Ltd. (Suzhou, China) at a laboratory temperature of 28.5°C. 5 dpf wild‐type AB line zebrafish and normally developing zebrafish under a microscope were selected for experiments.

The different groups and treatments were set as follows: (1) blank group (Ctrl): normal rearing, untreated; (2) model group (Model): placed in a light incubator with a 4000 lux illumination setting for continuous illumination for 24 h; (3) γ‐aminobutyric acid (GABA) (Huaxi Biotechnology Co. Ltd., Jinan, China) treatment group: zebrafish were treated with GABA aqueous administration for 24 h on a model basis; (4) estazolam (Shandong Xinyi Pharmaceutical Co. Ltd., Dezhou, China) treatment group (Estazolam): zebrafish were treated with aqueous administration of estazolam for 24 h on a model basis; (5) semen zizyphi spinosa treatment group (SZSP, SZSPE, SZSPE.βG): on the basis of the model, zebrafish were treated with aqueous administration of SZSP, SZSPE, and SZSPE.βG for 24 h respectively.

All animal experiments in this study were approved by the Ethics Committee of the Experimental Animal Center of Qilu University of Technology (Shandong Academy of Science) (Jinan, China) and strictly complied with the egulations on the Management of Laboratory Animalsissued by the Ministry of Science and Technology of the People's Republic of China.

### 2.4 Determination of the Maximum Tolerance Concentration (MTC) of Zebrafish Juveniles to Drugs

Wild‐type AB strain zebrafish were selected to be bred by natural pair mating, 450 fishes were randomly selected, with 30 fishes in each experimental group, and kept in E3 medium (1.74% NaCl, 0.29% CaCl2·2H2O, 0.08% KCl, 0.489% MgCl·6H2O and water) at 28.5°C in a light‐constant incubator (light/dark:14 h/10 h). Two days' zebrafish embryos after fertilization were treated with a gradient concentration of the drugs for 96 h. During the treatment, the survival rate, malformation, and heart rate of zebrafish embryos were observed and recorded every 24 h. SZSPE.βG was set at five concentrations of 5, 25, 50, 100, and 200 mg/mL. The positive drugs GABA and estazolam were set at five concentrations of 0.05, 0.1, 0.5, 1, and 5 mg/mL, respectively, and the concentrations of the treatments used in the subsequent experiments were determined according to the results of the experiments.

### 2.5 Behavioral Experiments on Zebrafish

Drug‐treated 5 dpf zebrafish juveniles were subjected to perform the behavioral experiments of behavioral waking activity volume at 10:00 a.m. at 6 dpf. The concentrations of each group were set as follows: GABA group 2 mg/mL (1/3 MTC), Estazolam group 2 mg/mL (1/3 MTC), SZSP group 0.1 mg/mL (MTC), SZSPE group 0.1 mg/mL (MTC), and SZSPE.βG group 0.1 mg/mL (MTC). Each group of juvenile fishes was placed into a 48‐well plate, with one fish in each hole and eight fishes in each group for a total of six groups, and the 48‐well plate was placed into the zebrafish behavior tester. After being placed in the dark enclosure of the behavioral testing apparatus, the juveniles were allowed to adapt for 15 min, and data were collected through the behavior analyzer in a quiet environment. The experiment lasted until 9:00 a.m. on the third day, with dark conditions from 11:00 p.m. to 9:00 a.m. and 50% light from 9:00 a.m. to 11:00 p.m. Data were collected at 10 min intervals. The maximum detection threshold (2000) and the minimum detection threshold were set(2). The test was performed after setting the behavioral parameters, and the data were output after 48 h of continuous monitoring.

### 2.6 Determination of GABA, Hypocretin/Orexin (HCRT), Neuromedin U (NMU) Content

5 dpf zebrafish juveniles treated with drugs were collected at 2:00 pm at 6 dpf, with 3 replicates per group and 50 fish per replicate. The content of GABA, HCRT, and NMU in zebrafish tissues was measured using ELISA kits (Shanghai Kexing Trading Co. Ltd., Shanghai, China).

### 2.7 RNA Extraction and Real‐Time Fluorescence Quantitative PCR Analysis

6 dpf zebrafish juveniles were sampled at 2:00 pm, with 30 samples taken from each group. Total RNA was extracted from the tissues using the RNA rapid extraction kit, and cDNA was generated by reverse transcription using a reverse transcription kit (Shandong Sparkjade Biotechnology Co. Ltd., Jinan, China). cDNA was diluted 20‐fold and used as an amplification template, and *β‐actin* was used as an internal parameter. The mRNA expression of GABA receptors (*gabra1*, *gabra5*, *gabrb2a*), *hcrt*, and *nmu* was quantified by real‐time PCR (QuantStudio 3 Real‐Time RCR Systems, Thermo Fisher, USA) using a fluorescence quantitative PCR kit (Shandong Sparkjade Biotechnology Co. Ltd., Jinan, China). The primers used for each gene were: *gabra1* forward 5′‐AACACCACAGTGTTCACCAG‐3′, reverse 5′‐CATGTCGTGGTCTGAAACTG‐3′; *gabra5* forward 5′‐AGAGTCAGAGCTCAATGATA‐3′, reverse 5′‐GGTGACAAAGATGTTGGTCT‐3′; *gabrb2a* forward 5′‐AGTCCTTCGTTCACGGAGTG‐3′, reverse 5′‐GTTCTGCTCGTCCAGCGGGT‐3′; *hcrt* forward 5′‐ATGGACTGCACAGCTAAG‐3′, reverse 5′‐CATCTCGTAGAGTTTGCAGG‐3′; *nmu* forward 5′‐ACGCGCAACCGCTCACAGCG‐3′, reverse 5′‐CTGCTCATGCTCTAGTGAAG‐3′; *β‐Actin* forward 5′‐CCCTGAATCCCAAAGCCAAC‐3′, reverse 5′‐TACAGAGAGAGCACAGCCTG‐3′. The PCR amplification parameters were as follows: (1) 95°C for 5 min, (2) 95°C for 20 s, (3) 60°C for 20 s, (4) 72°C for 20 s, and (5) repeat steps 2–4 for 40 cycles at 72°C for 10 min.

### 2.8 Statistical Analysis

Statistical analysis was conducted using SPSS 26.0 software. All data were displayed as mean ± standard deviation (SD). Analysis of variance (ANOVA) was performed by one‐way ANOVA, and differences between groups were calculated using LSD and Duncan's method. *p* < 0.05 or *p* < 0.01 were considered statistically significant.

## 3 Results

### 3.1 Total Jujuboside Content and Liquid‐Quality Target Quantification of JuA and JuB of SZSP, SZSPE, and SZSPE.βG

**FIGURE 1:**
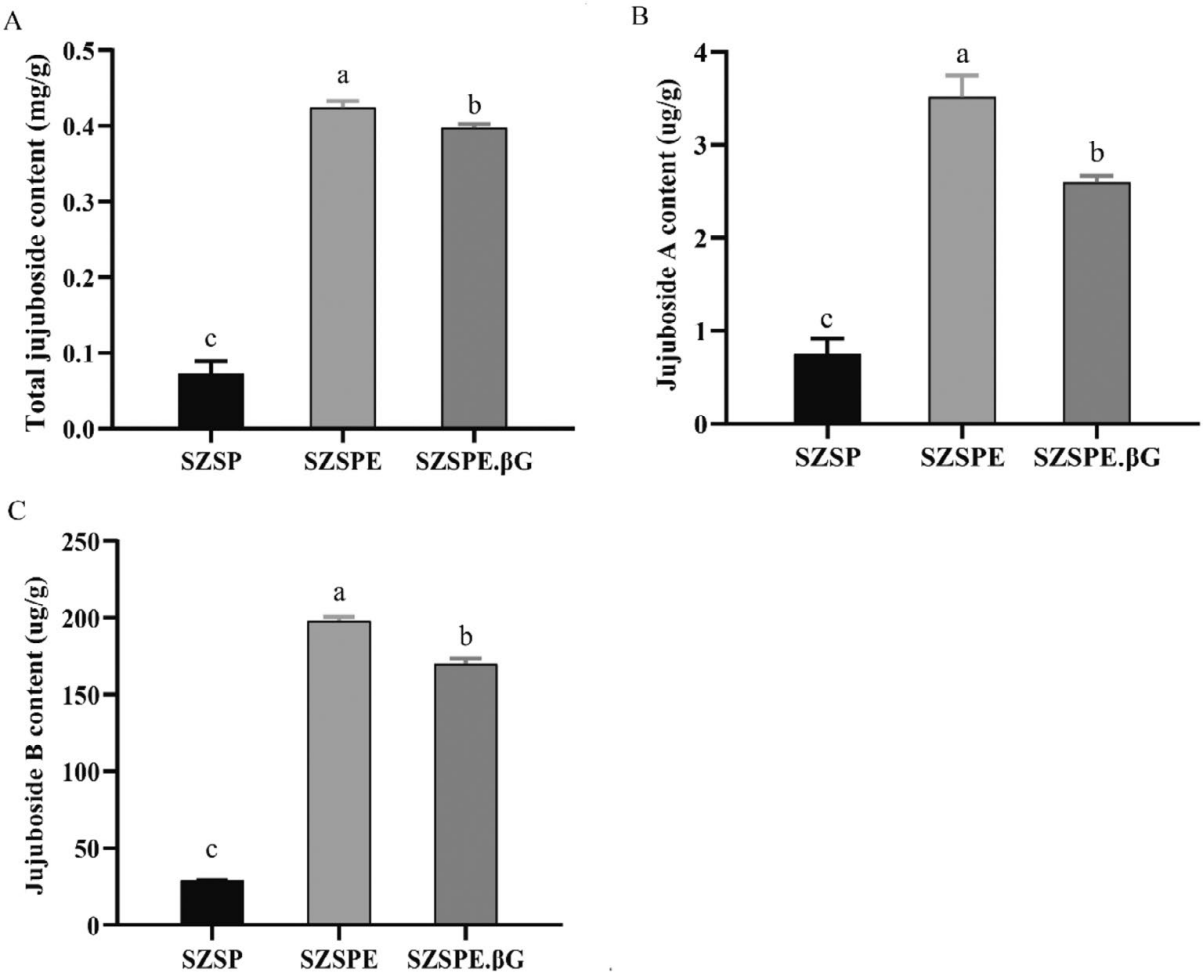
Changes of jujuboside content of semen ziziphi spinosae in different treatments. (A) Total jujuboside; (B) JuA; (C) JuB. Data are expressed as average ± SD (*n* = 3). The same superscript letter for the same indicator shows no significant difference, *p* > 0.05; different superscript letters indicate a significant difference, *p* < 0.05.

As shown in Figure 1A, the total jujuboside content of the SZSPE group was significantly increased by 482.14% (*p* < 0.01) compared to the SZSP group after ultrasonic assisted alcohol extraction, while the total jujuboside content of SZSPE.βG group after β‐glucosidase hydrolysis of SZSP decreased by 6.34% (*p* < 0.05) compared to the SZSPE group.

Based on the UHPLC‐QQQ‐MS detection platform, JuA and JuB liquid–liquid target quantification was performed on different treatments of semen ziziphi spinosae. JuA content was significantly increased by 366.81% (*p* < 0.01) in the SZSPE group compared to the SZSP group (Figure 1B). The peak of the mass spectrogram of JuA was significantly elevated in the SZSPE group (Figure S1A,B). Compared with the SZSPE group, the JuA content in the SZSPE.βG group was significantly reduced by 26.16% (*p* < 0.05) (Figure 1B), and the peak value of the mass spectrometry of JuA in SZSPE.βG group decreased, indicating that β‐glucosidase hydrolyzed part of the JuA and broke the glycosidic bond of part of the JuA, which led to a decrease in its content (Figure S1B,C). JuB content was significantly increased by 580.41% (*p* < 0.01) in the SZSPE group compared to the SZSP group (Figure 1C). Mass spectrometry analysis also confirmed a significant increase in the peak mass spectrogram of JuB in the SZSPE group (Figure S1D,E). Compared with the SZSPE group, the JuB content in the SZSPE.βG group was significantly reduced by 13.97% (*p* < 0.01) (Figure 1C), and the peak value of the mass spectrometry of JuB in SZSPE.βG group was decreased, which indicated that β‐glucosidase could hydrolyze JuB and break some of the glycosidic bonds of JuB, thereby reducing its content (Figure S1E,F). The possible chemical reactions of JuA and JuB in semen ziziphi spinosae when treated with β‐glucosidase are shown in Figure S2.

### 3.2 Maximum Tolerated Concentration (MTC) of Drugs in Zebrafish Juvenile

**FIGURE 2:**
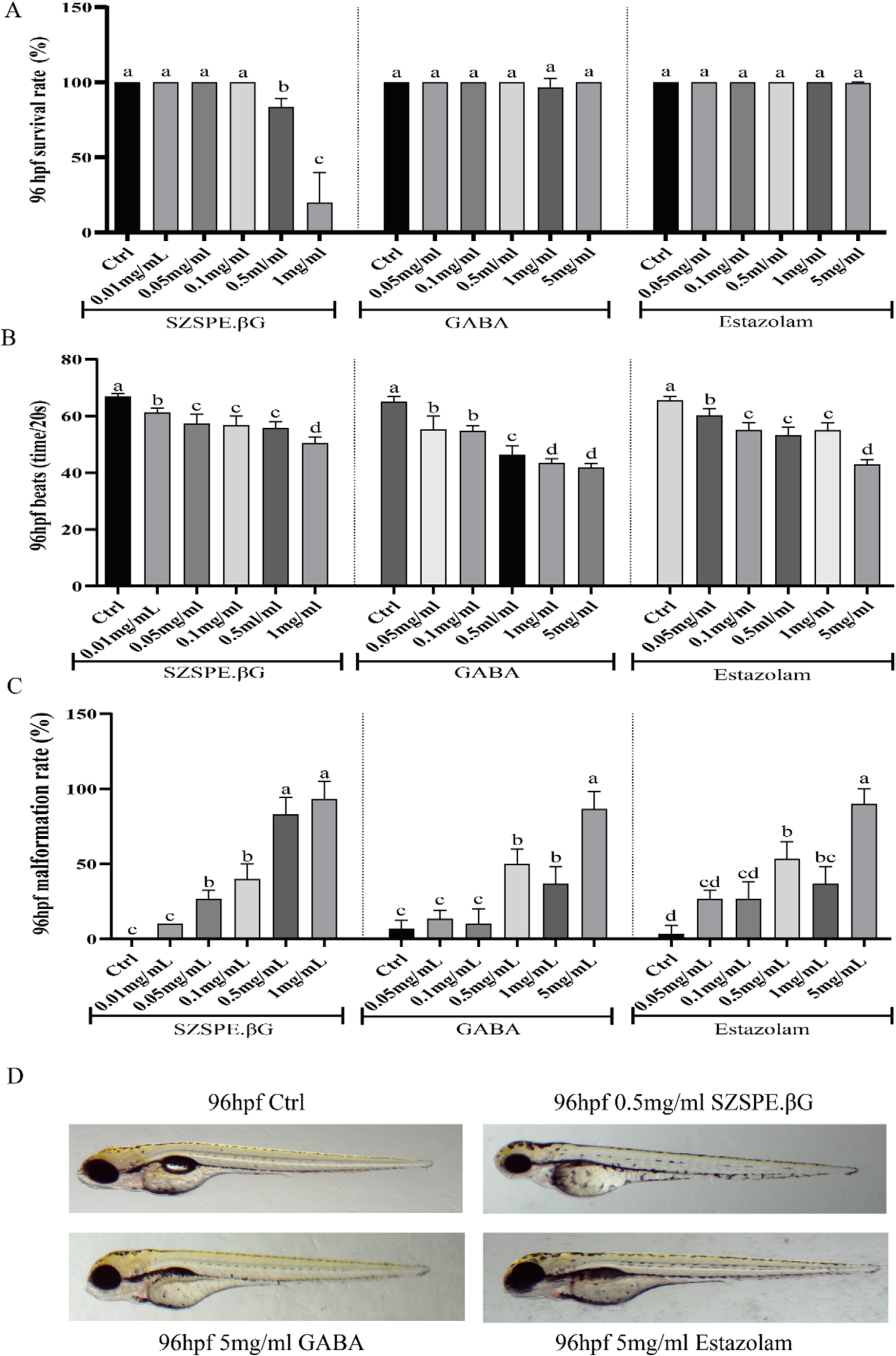
Survival rate, beats, and malformation rate of zebrafish juveniles treated with different samples. (A) Survival rate of zebrafish juveniles at 96 hdf stage treated with gradient concentrations of SZSPE.βG ABA and Estazolam. (B) Beats of zebrafish juveniles at 96 hdf stage treated with gradient concentrations of SZSPE.βG GABA and Estazolam. (C) Malformation rate of zebrafish juveniles at 96 hdf stage treated with gradient concentrations of SZSPE.βG GABA and Estazolam. (D) Malformations of zebrafish juveniles at 96hdf stage treated with different drugs. Data are expressed as average ± SD. The same superscript letter for the same indicator indicates no significant difference, *p* > 0.05; different superscript letters indicate a significant difference, *p* < 0.05.

As shown in Figure 2A–D, after the gradient treatment of SZSPE.βG, zebrafish juveniles in the 0.5 and 1 mg/mL groups at the 96 hdf stage appeared to die, with a mortality rate of 16.67% and 80%, respectively (*p* < 0.01). The heart rate of each treatment group was significantly lower than that of the Ctrl group (*p* < 0.01). The main malformation observed during sample treatment was undeveloped swim sacs, with a higher proportion of undeveloped juvenile fish in the high concentration treatment group. Therefore, MTC for SZSPE.βG was determined to be 0.1 mg/mL. After gradient treatment of GABA and estazolam, there was almost no death of zebrafish juveniles in each concentration treatment group during the 96 hdf stage. The heart rate was significantly lower in the treatment groups at all concentrations than that in the Ctrl group (*p* < 0.01). The teratogenic effect of this treatment group was relatively low, mainly due to the undeveloped sacs during the 96 hpf stage (*p* < 0.05). As a consequence, MTC for GABA and estazolam was determined to be 5 mg/mL, respectively.

### 3.3 Effects of SZSP, SZSPE, and SZSPE.βG Treatments on Zebrafish Waking Activity Levels

**FIGURE 3:**
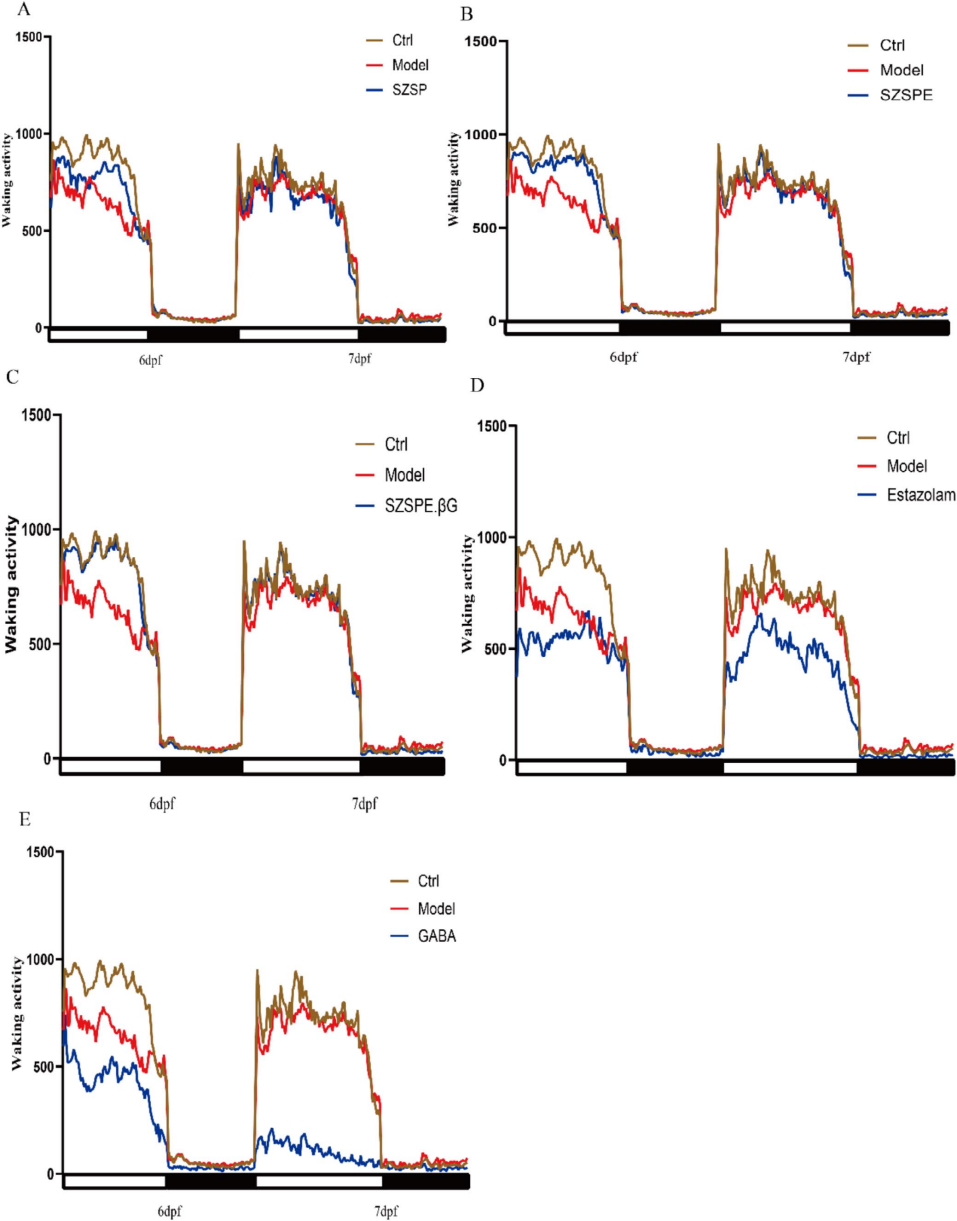
Time series analysis of waking activity. Calculated the average amount of awakening activity every 10 min, the black and white modules in the horizontal axis represent night and day, respectively. (A) SZSP; (B) SZSPE; (C) SZSPE.βG; (D) Estazolam; (E) GABA. Data are expressed as average ± SD. The same superscript letter for the same indicator indicates no significant difference, *p* > 0.05; different superscript letters indicate a significant difference, *p* < 0.05.

As depicted in Figure 3, the daytime waking activity of each zebrafish group was notably higher than their nighttime activity, indicating a pronounced circadian rhythm. During the daytime of the first day, the daytime waking activity in the Model group was significantly lower than that in the Ctrl group due to light deprivation sleep (*p* < 0.01). The waking activity in the SZSP, SZSPE, and SZSPE.βG groups was significantly higher than that in the Model group (*p* < 0.01), while the difference between SZSPE.βG and Ctrl groups was insignificant (*p* > 0.05) (Figure 3A–C). The waking activity of the GABA and Estazolam groups was significantly lower than that of the Model group (*p* < 0.01), which was consistent with their own side effect of drowsiness (Figure 3D,E); on the first night, the Model group had significantly higher nightly waking activity than the Ctrl group due to the sleep deprivation treatment (*p* < 0.01). Compared with the Model group, the SZSP and SZSPE groups showed a decrease in waking activity, but the difference was not significant (*p* > 0.05), and SZSPE.βG group had significantly lower waking activity than Model group (*p* < 0.01) (Figure 3A–C). The waking activity of the GABA and Estazolam group was significantly lower than that of the Model group (*p* < 0.01) (Figure 3D,E).

The above results indicated that the GABA group and Estazolam group have a significant improvement effect on zebrafish sleep, but their side effects of drowsiness were more obvious. The SZSP and SZSPE group had a certain improvement effect on zebrafish sleep. SZSPE.βG group had a significant improvement on zebrafish sleep and did not show the side effect of drowsiness.

### 3.4 Effects of SZSP, SZSPE, and SZSPE.βG Treatment on the Contents of Sleep‐Related Substances GABA, HCRT, and NMU in Zebrafish Tissues

**FIGURE 4:**
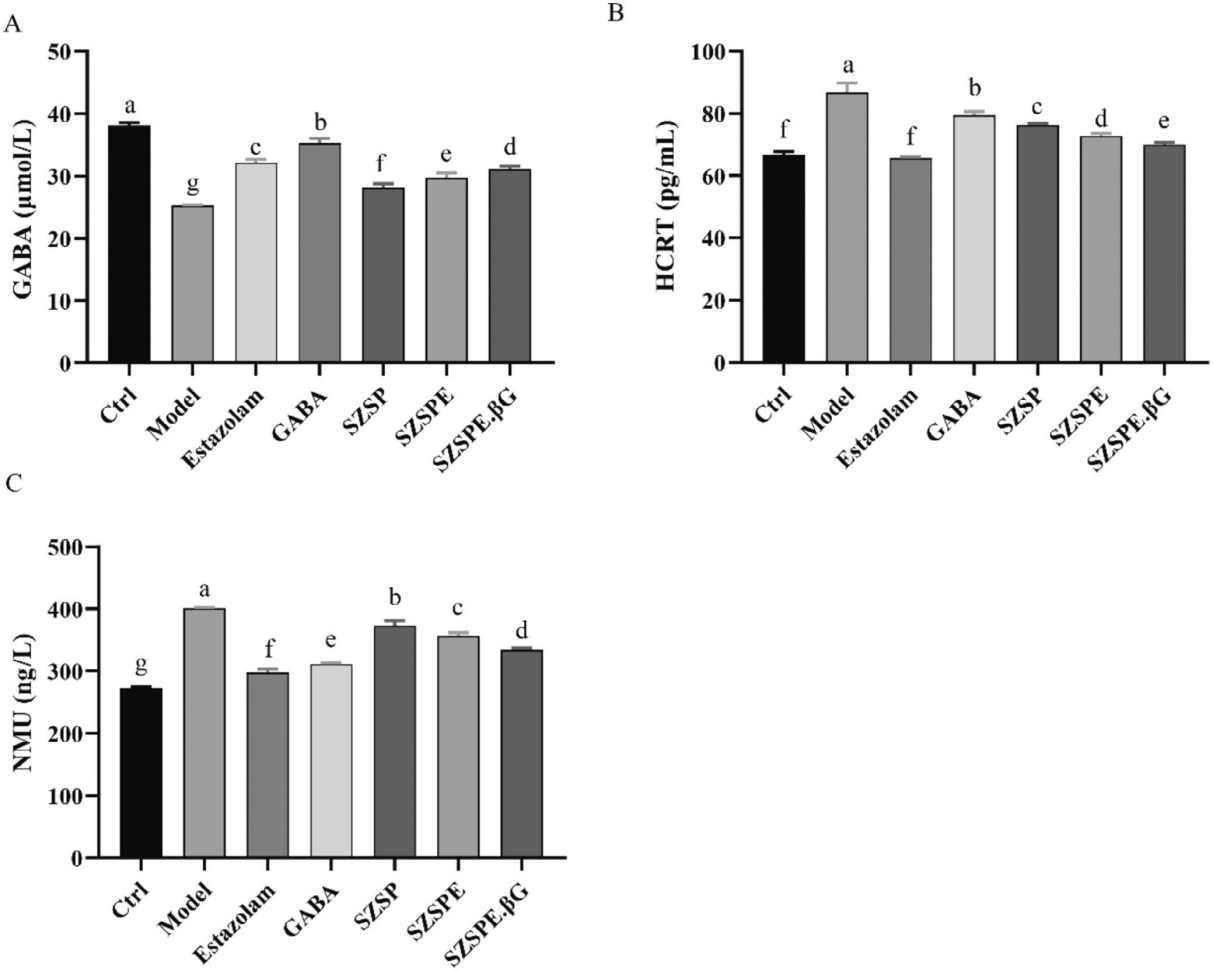
Changes of the content of sleep‐related substances. (A) GABA; (B) HCRT; (C) NMU. Data are expressed as average ± SD (*n* = 3). The same superscript letter for the same indicator indicates no significant difference, *p* > 0.05; different superscript letters indicate a significant difference, *p* < 0.05.

As shown in Figure 4A, compared with the Ctrl group, the Model group showed a significant decrease in GABA content by 33.78% (*p* < 0.01) due to continuous light stimulation being in an excited state. Compared to the Model group, the GABA content was significantly increased by 11.43%, 17.92%, and 23.27% (*p* < 0.01) in SZSP, SZSPE, and SZSPE.βG groups, respectively, while the Estazolam and GABA groups significantly increased by 27.48% and 39.80%, respectively (*p* < 0.01). HCRT content was significantly increased by 29.84% in the Model group compared to the Ctrl group (*p* < 0.01). Compared with the Model group, HCRT was significantly reduced by 12.01%, 15.96%, 19.19%, 24.26%, and 8.33% in SZSP, SZSPE, SZSPE.βG, Estazolam, and GABA groups, respectively (*p* < 0.01), indicating that GABA did not primarily improve sleep by regulating HCRT (Figure 4B). As shown in Figure 4C, the NMU content in the Model group was significantly increased by 46.81% (*p* < 0.01) compared to the Ctrl group. Compared to the Model group, the NMU content was significantly reduced by 6.87%, 11.07%, 16.60%, 25.72%, and 22.30% (*p* < 0.01) in SZSP, SZSPE, SZSPE.βG, Estazolam, and GABA groups, respectively, and by 25.72% and 22.30% (*p* < 0.01) in the Estazolam and GABA groups, respectively.

### 3.5 Effects of SZSP, SZSPE, and SZSPE.βG Treatment on the Expression of Sleep‐Related Genes

**FIGURE 5:**
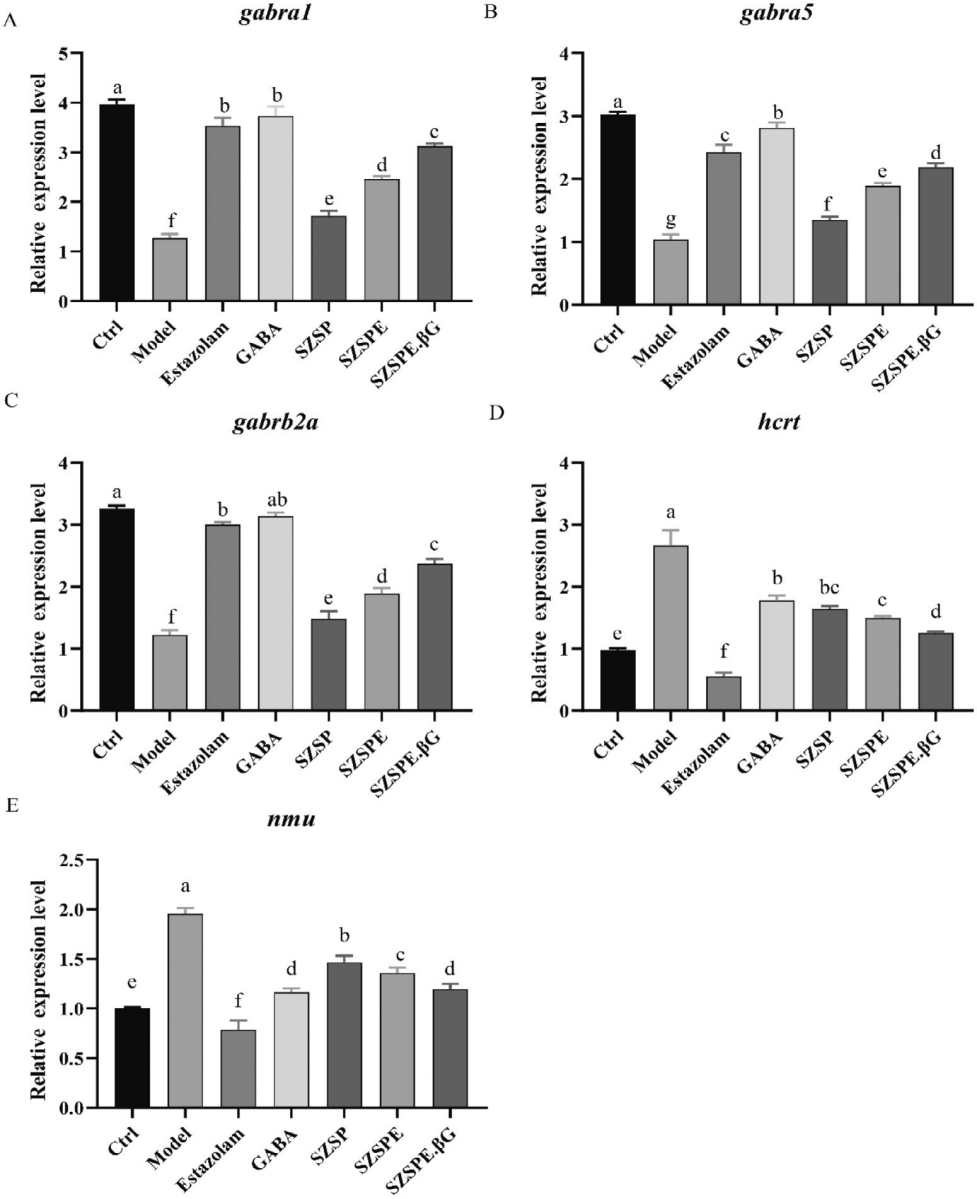
Effects of different treatments on mRNA transcript levels of sleep‐related genes in zebrafish. (A) *gabra1*; (B) *gabra5*; (C) *gabrb2a*; (D) *hcrt*; (E) *nmu*. Data are expressed as average ± SD (*n* = 3). The same superscript letter for the same indicator indicates no significant difference, *p* > 0.05; different superscript letters indicate a significant difference, *p* < 0.05.

As shown in Figure 5A–C, compared with the Ctrl group, the relative mRNA expression level of *gabral1*, *gabral5*, and *gabrb2a* in zebrafish tissues of the Model group was significantly reduced (*p* < 0.01). Compared with the Model group, the relative mRNA expression level of *gabra1*, *gabra5*, and *gabrb2a* was significantly increased in all treatment groups (*p* < 0.01), among which the relative mRNA expression levels of *gabra1*, *gabra5*, and *gabrb2a* in the SZSP, SZSPE, and SZSPE.βG groups were significantly lower than those in the Estazolam and GABA groups (*p* < 0.05), while the relative expression levels of *gabra1*, *gabra5*, and *gabrb2a* in SZSPE.βG group were significantly higher than those in the SZSP and SZSPE groups (*p* < 0.01). The above results indicated that each treatment could improve the sleep of zebrafish by up‐regulating the expression level of mRNA of the GABA receptor.

It was found that the relative mRNA expression level of *hcrt* was significantly higher in the Model group compared to the Ctrl group (*p* < 0.01), suggesting that overexpression of *hcrt* increased the autonomous activity of zebrafish. Compared with the Model group, the relative mRNA expression level of *hcrt* in each treatment was significantly reduced (*p* < 0.01). The relative mRNA expression level of *hcrt* in SZSPE.βG group was significantly reduced compared to the GABA group (*p* < 0.01) and significantly increased compared to the Estazolam group (*p* < 0.01). The SZSP and SZSPE groups showed a decrease compared to the GABA group, but the difference was not significant (*p* > 0.05), and they were significantly higher than the Estazolam group (*p* < 0.01) (Figure 5D).

As shown in Figure 5E, the relative mRNA expression level of *nmu* was significantly higher in the Model group compared to the Ctrl group (*p* < 0.01), suggesting that the overexpression of *nmu* led to an increase in autonomous activity in zebrafish. Compared with the Model group, the relative mRNA expression level of *nmu* in each treatment was significantly reduced (*p* < 0.01). The relative mRNA expression level of *nmu* was significantly higher in the SZSP and SZSPE groups compared with the Estazolam and GABA groups (*p* < 0.01, *p* < 0.05), while SZSPE.βG group showed a significant increase compared to the Estazolam group (*p* < 0.01), but there was no significant difference when compared to the GABA group (*p* > 0.05).

## 4 Discussion

Although various clinical and pharmacological experiments have confirmed that jujuboside is the main effective ingredient of semen ziziphi spinosae in calming and hypnotic effects, their large molecular weight and complex chemical structure limit their application in sedative‐hypnotic and neurological disorders, with only a small portion being converted and absorbed to exert their efficacy (Chen and Huang 2011). Saponins, as the largest class of natural plant products, have complex compositions and are highly polar, sugar‐containing compounds. The intricate sugar chains structure makes it difficult to hydrolyze sugar groups, resulting in a decrease in the lipid solubility of saponin analogues, difficulty in being absorbed effectively by small intestinal villi, and low bioavailability. However, upon the removal of sugar groups, the polarity of secondary glycosides and saponin glycosides decreases, while their lipid solubility increases. Consequently, they can be swiftly absorbed into the bloodstream through the small intestine after ingestion (Augustin et al. 2011; Silberberg et al. 2006). Therefore, the conversion of natural saponins into saponin glycosides with higher bioactivity and bioavailability through biotransformation is a cost‐effective and efficient way to enhance the effectiveness of their application in insomnia, neurological disorders, and other fields. The biological enzyme method offers significant advantages in terms of specificity, conversion rate, and preserving the core structure of saponins. Presently, β‐glucosidase has been the most extensively researched saponin bioconversion enzyme and has been successfully employed in removing sugar groups from ginsenosides (Otieno et al. 2006; Pei et al. 2015). β‐Glucosidase is a glycoside hydrolase that acts on glycosidic bonds in the carbohydrate fraction, releasing non‐reducing terminal glycosyl residues, glycosides, and oligosaccharides. Studies have shown that β‐glucosidase is capable of hydrolyzing glycosidic compounds and thereby producing aglycones with heightened biological activity (Hati et al. 2015; Ruviaro et al. 2019). Currently, no studies have been reported on the application of β‐glucosidase in the extraction and conversion of jujuboside.

In this study, our investigation revealed a significant increase in the total jujuboside content in semen ziziphi spinosae after ultrasonic alcohol extraction (Figure 1A). Ultrasonic extraction utilized ultrasonic waves to generate a high‐speed, powerful cavitation effect and agitation to disrupt the cell wall structure of medicinal plant herbs, so that the drug components in plant cells can be released and fully mixed with the solvent, thus improving the extraction rate (Liu et al. 2018). Hydrolysis of the above extracts by β‐glucosidase resulted in a significant decrease in the total jujuboside content of SZSPE.βG as compared to SZSPE (Figure 1A). The reason for this is that β‐glucosidase hydrolyzes and breaks the glycosidic bond connecting two glucose or glucose‐substituted molecules, removes the non‐reducing terminal glucosyl residues in sugars and glycosides (Ketudat Cairns and Esen 2010), thereby converting some jujuboside into secondary saponins or jujubogenin. UHPLC‐QQQ‐MS assay found that the contents of JuA and JuB were significantly decreased after hydrolysis by β‐glucosidase (Figure 1A,B). JuA and JuB both consist of different amounts of glycosidic bonds, and the product of JuA's metabolic hydrolysis by gut microbiota is JuB, both of which are the main active substances of semen ziziphi spinosae for improving sleep (Liu et al. 2021). In this study, it was found that the glycosidic bonds in JuA and JuB were hydrolyzed and broken by β‐glucosidase, resulting in a significant increase in the content of jujubogenin devoid of glycosidic bonds (Figures 1 and S2). These findings suggested that after hydrolysis by β‐glucosidase, jujuboside was partially converted into jujubogenin, which exhibits higher biological activity and enhanced absorption properties.

Zebrafish predominantly exhibit sleep behavior during nighttime hours, displaying a well‐defined circadian rhythm. Exposure to continuous light for a period of 3 days led to a substantial suppression of zebrafish sleep, with nearly 90% reduction, followed by a gradual recovery over a span of 12 weeks. This research used a 24‐h light‐induced sleep deprivation model in zebrafish, which is a better sleep deprivation model compared to other modeling approaches (Yokogawa et al. 2007). Behavioral monitoring showed that on the first and second night after administration, SZSP, SZSPE, and SZSPE.βG groups effectively increased the sleep of zebrafish juveniles compared to the Model group. Concurrently, these treatments exhibited an increase in waking activity on both the first and second days, when compared to the Model group (Figure 3). It was reported that semen ziziphi spinosae significantly prolonged the total sleep time in a rat model (Zhang et al. 2014), suggesting that the sleep‐aiding activity of semen ziziphi spinosae extract is conserved among zebrafish juveniles and mammals. In addition, our study found that while promoting sleep, semen ziziphi spinosae extract significantly increased the daytime waking activity of zebrafish juveniles, resulting in a more regular and significant circadian rhythm. SZSPE.βG group was significantly more effective in improving the sleep of zebrafish juveniles than the SZSP and SZSPE groups, suggesting that the jujubogenin in semen ziziphi spinosae has a higher bioavailability for sleep‐deprived zebrafish. The increased effectiveness can be attributed to the higher content of active jujubogenin following β‐glucosidase hydrolysis. When the SZSP extract was introduced into zebrafish, it primarily contained glycosides, which existed in the form of prodrugs and are challenging to absorb in the intestinal tract. However, after hydrolysis by β‐glucosidase, these glycosides were converted into jujubogenin, thereby improving lipid solubility. This transformation allows for rapid absorption into the bloodstream through the small intestine after ingestion, thus achieving the required blood concentration faster (Silberberg et al. 2006). Consequently, as previous studies have shown, this will result in a more potent sleep‐aid effect. It is worth noting that although the GABA and Estazolam groups significantly improved the sleep of zebrafish juveniles, their daytime waking activity also decreased, which is consistent with the known side effects of benzodiazepine drugs, such as drowsiness (De Crescenzo et al. 2022). In contrast, the extract of semen ziziphi spinosae has minimal impact on the daytime functioning of zebrafish juveniles, making it a potentially more favorable option for sleep enhancement without interfering with daytime activities.

GABA is a major inhibitory neurotransmitter in the central nervous system, playing a decisive role in blocking the conduction of excitability and sleep regulation. Numerous studies have shown that the hypnotic effect of some drugs is to enhance the binding of GABA to recognition sites by increasing the affinity of GABA receptors. Some drugs can increase the content of GABA in the brain by inhibiting its decomposition, which to some extent increases slow‐wave sleep time (Olsen and Sieghart 2009; Yang et al. 2005). Monkeys continuously stimulated by excitation have significantly lower levels of GABA in their brain tissues compared to normal monkeys, and increasing the GABA content in the brain can effectively improve neurological function (Leventhal et al. 2003). In this study, we found that zebrafish juveniles of the model group were in an excited state due to continuous light exposure, and their GABA content was significantly lower than the Ctrl group. SZSP, SZSPE, and SZSPE.βG groups can effectively increase the content of GABA in zebrafish tissues (Figure 4A), indicating that the saponin molecules (including jujuboside and jujubogenin) can effectively increase the content of GABA in zebrafish tissues, enhance the inhibitory effect on excitatory synaptic transmission, and achieve sedative and hypnotic effects. The action of GABA is mediated by ligand‐gated ion channels through GABA_A_ and GABA_C_ receptors, and by GABA_B_ receptors coupled with Gi proteins that promote potassium ion conductance upon activation. Among them, the GABA_A_ receptor is a widely used target for benzodiazepine and nonbenzodiazepine drugs in clinical practice. When GABA binds to the GABA_A_ receptor, the channel opens and chloride ions flow into the cell along the concentration gradient, causing an increase in the negative charge in the neuronal cell and making it more difficult to stimulate an action potential, thereby achieving inhibition (Richey and Krystal 2011). Studies have shown that jujuboside can achieve hypnotic effects by regulating the expression of various subtypes of GABA_A_ receptors in the hippocampus of rats in vitro (You et al. 2010). In our study, we observed the significant upregulation of mRNA expression of the α1, α5, and β2 isoforms of GABA receptors in zebrafish tissues following the administration of SZSP, SZSPE, and SZSPE.βG (Figure 5A–C), indicating that jujuboside not only enhances the inward flow of chloride ions by binding to GABA receptors but also triggers the overexpression of GABA receptors, thereby prolonging sleep time (Han et al. 2009). Among them, the SZSPE.βG group was particularly effective in regulating both GABA content and GABA receptor mRNA expression levels, which also indicates that, relative to JuA and JuB, jujubogenin has a higher blood–brain barrier permeability and a high binding affinity for the GABA_A_ receptor (C. Y.‐C. Chen 2009).

The HCRTergic system is one of the major neural networks regulating sleep/wakefulness in mammals besides the GABAergic system. Numerous studies have demonstrated that HCRT mediates arousal in zebrafish, similar to that in mammals. Overexpression of HCRT neurons with arousal activity in zebrafish juveniles can lead to increased motor activity and decreased sleep. In contrast, the ablation of HCRT neurons leads to increased sleep (Elbaz et al. 2013; Prober et al. 2006). It was found in this study that SZSP, SZSPE, and SZSPE.βG groups effectively reduced HCRT levels and down‐regulated the mRNA expression level of *hcrt* in the tissues of zebrafish juveniles (Figures 4B, 5D), indicating that jujuboside and jujubogenin extracted from semen ziziphi spinosae may achieve sedative‐hypnotic effects by down‐regulating the level of HCRT neurons. In contrast, the GABA group did not significantly influence HCRT neurons, which may be due to the fact that HCRT neurons do not belong to the GABAergic system (Du and Du 2015).

NMU is a neuropeptide modulator of sleep/awakening in mammals, widely distributed in the nervous system and tissues and organs. It has been shown that the expression of *nmu* mRNA in rats varies day and night; overexpression of NMU increases its autonomic activity, thereby reducing sleep (Chiu et al. 2016; Honzawa et al. 1987), which is consistent with the results of this study. In the present study, zebrafish juveniles of the model group showed a significantly increased NMU expression level in their tissues as a result of continuous light stimulation and central activation of NMUR2 receptors, thus leading to insomnia. The levels of NMU and its mRNA expression were significantly down‐regulated in the tissues of zebrafish juveniles in SZSP, SZSPE, and SZSPE.βG groups (Figures 4C, 5E), suggesting that jujuboside and jujubogenin extracted from semen ziziphi spinosae may improve sleep by down‐regulating the levels of NMU to reduce the nocturnal voluntary activities of zebrafish juveniles.

The results of this study offer a theoretical basis for the utilization of semen ziziphi spinosae extract, as well as the extract enriched with jujubogenin through the hydrolysis of jujuboside using β‐glucosidase, in the context of sedation, hypnosis, and the prevention and treatment of neurological disorders. Semen ziziphi spinosae extract and the jujubogenin‐enriched extract hold promising potential as candidates or associated functional foods for addressing neurological conditions, including insomnia, thereby carrying significant therapeutic value.

## Author Contributions

**Wanxia Wang:** methodology, writing – original draft. **Can Li:** conceptualization, methodology, writing – original draft, writing – review and editing. **Lu Shen:** investigation. **Yifan Liu:** investigation. **Jie Zhou:** investigation. **Chengyun Zheng:** validation. **Dongqi Tang:** validation. **Fang Xiao:** conceptualization, validation. **Tao Xia:** conceptualization, writing – review and editing, resources.

## Conflicts of Interest

The authors declare no conflicts of interest.

## Supporting information


**Figure S1.** Mass spectra of JuA and JuB of different treatment groups.

**Figure S2.** Possible chemical reactions of JuA and JuB in semen ziziphi spinosae after β‐glucosidase hydrolysis.

## Data Availability

All datasets generated for this study are included in the article/Supporting Information.
