# Supplementary material for: Sleep Aid Effect and Mechanism of Semen Zizyphi Spinosae Extract Enriched With Jujuboside and Jujubogenin in Sleep‐Deprived Zebrafish
Source: Food Sci Nutr. 2025 Jun 11;13(6):e70413. doi: 10.1002/fsn3.70413 (PMC12158664; doi:10.1002/fsn3.70413)
Supplement: Supplementary file 1 — Figure S1. Mass spectra of JuA and JuB of different treatment groups. [file FSN3-13-e70413-s002.docx]

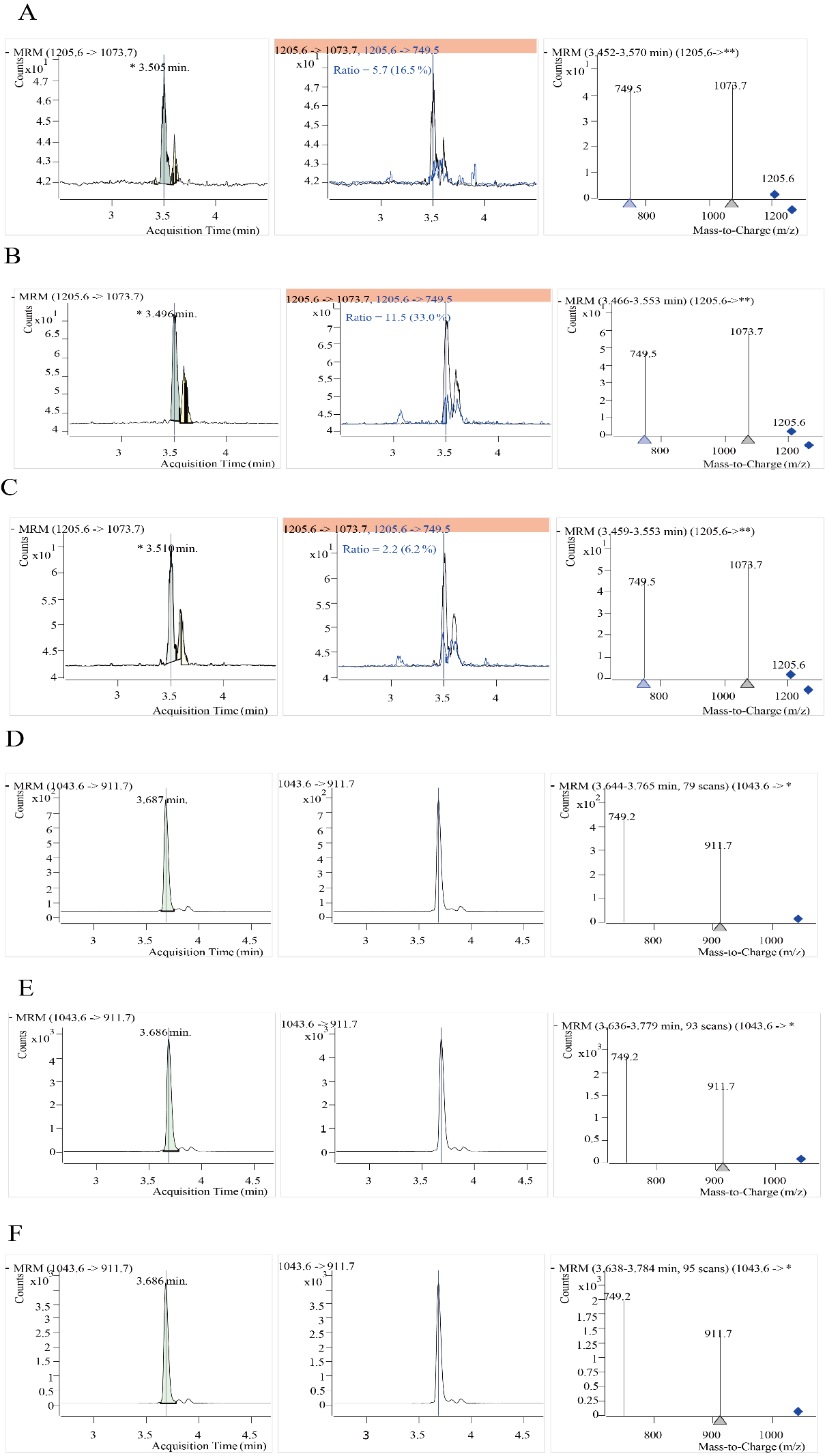


**Fig. S1.** Mass spectra of JuA and JuB of different treatment groups. (A) Mass spectra of JuA in SZSP; (B) Mass spectra of JuA in SZSPE; (C) Mass spectra of JuA in SZSPE.βG; (D) Mass spectra of JuB in SZSP; (E) Mass spectra of JuB in SZSPE; (F) Mass spectra of JuB in SZSPE.βG.
