# Supplementary material for: Sleep Aid Effect and Mechanism of Semen Zizyphi Spinosae Extract Enriched With Jujuboside and Jujubogenin in Sleep‐Deprived Zebrafish
Source: Food Sci Nutr. 2025 Jun 11;13(6):e70413. doi: 10.1002/fsn3.70413 (PMC12158664; doi:10.1002/fsn3.70413)
Supplement: Supplementary file 2 — Figure S2. Possible chemical reactions of JuA and JuB in semen ziziphi spinosae after β‐glucosidase hydrolysis. [file FSN3-13-e70413-s001.docx]

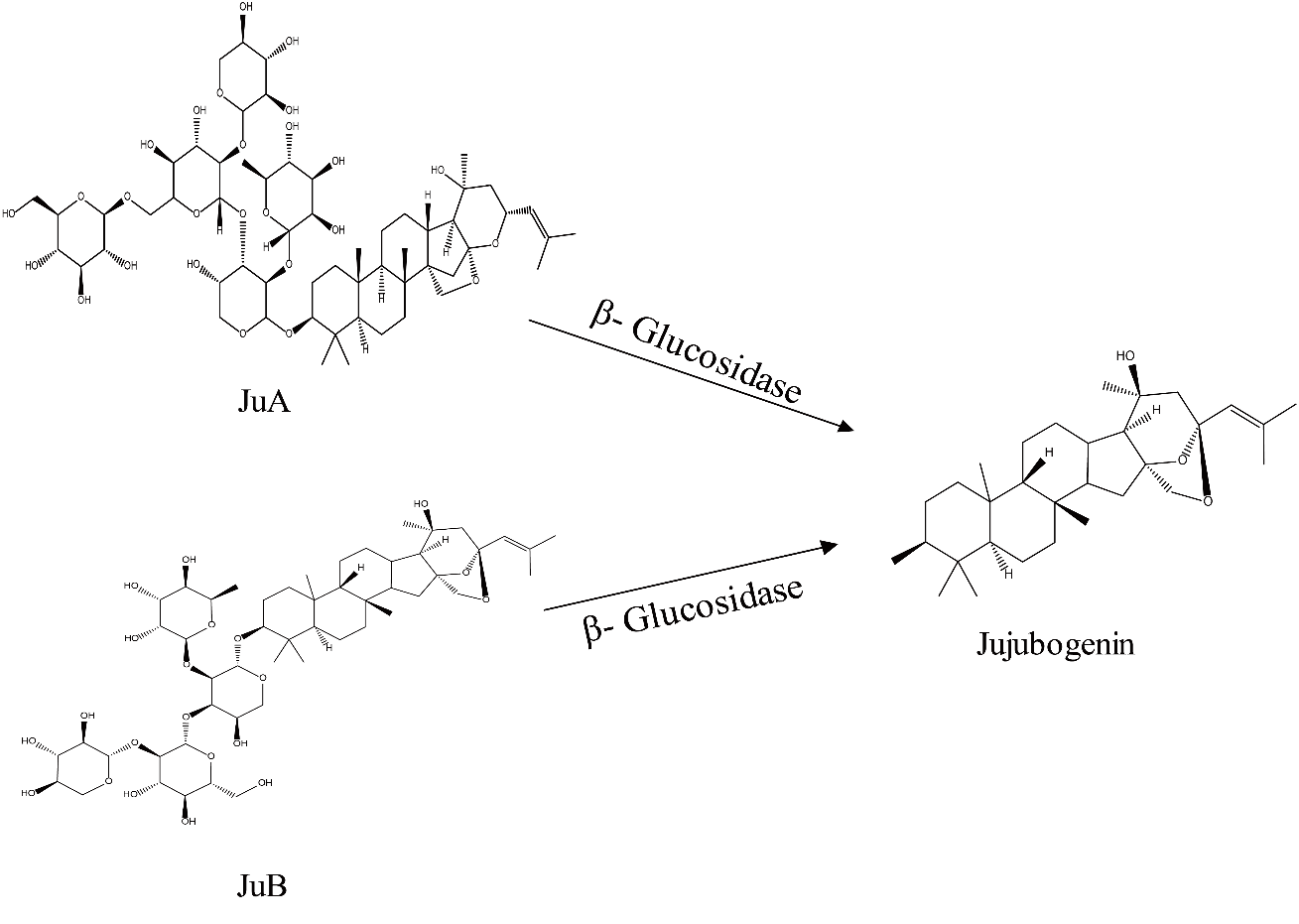


**Fig. S2.** Possible chemical reactions of JuA and JuB in semen ziziphi spinosae after β-glucosidase hydrolysis
